# RNA-Seq Analysis of Morphine-Induced Gene Expression Changes in the Mouse Nucleus Accumbens

**DOI:** 10.5812/ijpr-169453

**Published:** 2026-06-02

**Authors:** Seyed Mahmoud Pourmand, Shahrzad Nazari, Elham Nazari, Reza Arezoomandan, Gholamreza Hassanzadeh, Sahar Eshrati, Morteza Karami-Zarandi, Mohammad Bagher Saberi-Zafarghandi

**Affiliations:** 1 Department of Addiction, Tehran Institute of Psychiatry, School of Behavioral Sciences and Mental Health, Iran University of Medical Sciences, Tehran, Iran; 2 Department of Neuroscience and Addiction Studies, School of Advanced Technologies in Medicine, Tehran University of Medical Sciences, Tehran, Iran; 3 Proteomics Research Center, Faculty of Paramedical Sciences, Shahid Beheshti University of Medical Sciences, Tehran, Iran; 4 School of Behavioral and Brain Sciences, The University of Texas at Dallas, Richardson, USA; 5 Department of Anatomy, School of Medicine, Tehran University of Medical Sciences, Tehran, Iran; 6 Department of Digital Health, School of Medicine, Tehran University of Medical Sciences, Tehran, Iran; 7 Department of Microbiology, School of Medicine, Zanjan University of Medical Sciences, Zanjan, Iran

**Keywords:** Morphine, DESeq2, Nucleus Accumbens

## Abstract

**Background:**

Opioid use disorder (OUD) remains a critical global health challenge. The nucleus accumbens (NAc), a key region of the brain reward system, plays an essential role in opioid-induced neuroadaptations. Characterizing gene expression alterations in reward-system regions, such as the NAc, is essential for elucidating the molecular mechanisms underlying addiction. In recent years, bioinformatics has emerged as a rapidly advancing field and has played a pivotal role in elucidating molecular mechanisms and in advancing computational biology.

**Objectives:**

This study aimed to characterize transcriptomic changes in the NAc of mice following chronic morphine administration using RNA sequencing and bioinformatic analysis.

**Methods:**

RNA sequencing (RNA-Seq) data from morphine- and saline-treated mice were obtained from the Gene Expression Omnibus database via the Sequence Read Archive. Following quality control and alignment, differentially expressed genes (DEGs) were identified using the DESeq2 package in R. Gene Ontology (GO) and Kyoto Encyclopedia of Genes and Genomes (KEGG) enrichment analyses, as well as protein-protein interaction (PPI) network analyses, were performed to evaluate the functional implications of these genes.

**Results:**

Principal component analysis (PCA) revealed distinct transcriptomic profiles between the morphine and control groups. A total of 143 DEGs were identified, including 125 up-regulated and 18 down-regulated genes. Enrichment analyses indicated significant involvement in synaptic signaling, ion transport, and neurodegenerative pathways. Protein–protein interaction (PPI) network analysis identified several hub genes, including fgf3 (fibroblast growth factor 3), mki67 (marker of proliferation Ki-67), grin2a/grin2b (glutamate receptor NMDA subunits 2A/2B), gli1 (GLI family zinc finger 1), and ago2 (argonaute RISC catalytic component 2), which are associated with synaptic plasticity, neurogenesis, and epigenetic regulation.

**Conclusions:**

Chronic morphine exposure induces widespread gene expression changes in the NAc, engaging pathways associated with synaptic remodeling, neuronal excitability, and addiction-related neuroplasticity. These findings provide a molecular framework for understanding opioid-induced adaptations and identify candidate targets, including N-methyl-D-aspartate (NMDA) receptor subunits, fgf3, and ago2, for potential therapeutic intervention. Future studies should functionally validate these targets and evaluate their translational relevance in the context of OUD.

## 1. Background

Opioid use disorder constitutes a pressing global health concern, characterized by rising rates of dependence, adverse health outcomes, and mortality. Recent estimates indicate that millions of people worldwide are affected by opioid addiction, with a substantial disease burden reflected in increasing morbidity and mortality (1, 2). Understanding the molecular and genetic underpinnings of OUD is crucial for developing effective intervention strategies.

Conventional approaches to pain management frequently involve potent analgesics such as morphine, which is effective but has a high potential for abuse and dependence (3). Morphine addiction remains a significant challenge in pain management because of this elevated risk. The dual nature of morphine as both a therapeutic agent and a potential substance of misuse complicates its clinical use (4). Morphine dependence is a complex condition closely linked to the brain reward circuitry, including the NAc. The NAc, located in the ventral striatum, is a principal region in the reward circuit (5). Chronic morphine exposure alters gene expression and synaptic plasticity in the NAc and other regions of the reward system (6-8). Identifying the molecular and cellular underpinnings of these alterations in this region is essential for developing innovative therapeutic strategies.

In recent years, advances in bioinformatics and systems biology have transformed addiction research. These interdisciplinary fields enable the integration and analysis of large-scale molecular datasets, allowing the systematic identification of key genes, pathways, and networks involved in substance use disorders. Similar bioinformatics studies have provided valuable insights into the molecular mechanisms underlying addiction and have identified novel targets for intervention and therapy (9-11). Moreover, because biomarkers play important roles in addiction research, including facilitating drug development, improving diagnostic accuracy, and predicting relapse risk (12), such studies have considerable potential to identify promising biomarker candidates (9, 10).

The Gene Expression Omnibus (GEO) database is central to this approach, providing access to comprehensive transcriptomic profiles across diverse experimental conditions (13). By using public datasets from GEO, researchers can perform integrative analyses to uncover gene expression patterns and biological pathways that are responsive to opioid exposure.

## 2. Objectives

In the present study, we analyzed RNA-Seq data from the NAc of morphine-exposed mice and saline-treated controls, as curated in the GEO repository. Using robust bioinformatics approaches, including DEG analysis, GO analysis, pathway enrichment analysis, and PPI network analysis, we aimed to characterize morphine-induced molecular alterations within the NAc. These findings are expected to advance the molecular understanding of opioid addiction and may help identify key biological targets for future therapeutic development.

## 3. Methods

### 3.1. Raw Data Processing Method and Software Sources

RNA-Seq raw data from morphine- and saline-treated mouse groups in the study by Hofford et al. (14) were downloaded in FASTQ format from the Sequence Read Archive via GEO (www.ncbi.nlm.nih.gov/geo). Quality control and preprocessing of FASTQ files were performed to obtain clean data for downstream analyses. High-quality reads were obtained using Trimmomatic (15) (https://usegalaxy.org/). Transcriptome reads were then aligned to the mm10 reference file (16) using HISAT2 (17) (https://usegalaxy.org/), and count data were generated.

### 3.2. Preliminary Analysis of Gene Count Data, PCA, and DEGs

Principal component analysis is a statistical technique used to reduce data dimensionality, enabling simpler and more intuitive interpretation of data features. This method is typically applied when the number of features is large and the objective is to reduce model complexity or identify principal features. Normalized gene expression data, including log-transformed counts per million (CPM), were subjected to PCA to reduce dimensionality and visualize sample clustering based on global transcriptomic profiles. The first 2 principal components, PC1 and PC2, were plotted to assess sources of variance and group separation. PCA plots were generated using the ggplot2, plyr, and DESeq2 packages in R, focusing on genes exhibiting a significant mean difference as determined by analysis of variance (P ≤ 0.05) within the sample (9, 18).

The DESeq2 package was used to analyze RNA-Seq data and identify important and differentially expressed genes between groups using statistical models (19). By calculating metrics such as P values and expression-change thresholds, such as log2 fold change, DESeq2 can identify biologically important genes that are differentially expressed under different conditions. Genes with log2 fold change > 0 were considered up-regulated, and genes with log2 fold change < 0 were considered down-regulated. In this study, based on previous studies, genes with P < 0.05 and log2 fold change > 0.5 were classified as up-regulated genes, and genes with P < 0.05 and log2 fold change < -0.5 were classified as down-regulated genes (20). A volcano plot was generated using the DESeq2 package to display key genes that may be mechanistically important in disease. In this plot, genes on the left are down-regulated, and genes on the right are up-regulated. Genes whose expression was more altered in morphine-injected samples than in saline-injected samples were visualized using a heatmap generated with the pheatmap package in R.

### 3.3. GO and Pathway Enrichment Analysis

Using clusterProfiler in R, along with the org.Mm.eg.db annotation data from Bioconductor, GO analysis was performed on the differentially expressed genes (21). In this context, KEGG-based pathway enrichment analysis was performed using the clusterProfiler, org.Mm.eg.db, KEGGREST, enrichplot, and ggplot2 packages in R. Enrichment was evaluated across biological processes, molecular functions, and cellular components. Results were visualized using a category network plot (cnetplot) to depict relationships between DEGs and enriched GO terms, as well as dotplots summarizing the top GO terms and KEGG pathways. The size of each node or dot represents the number of associated genes, and the color indicates statistical significance.

### 3.4. PPI Network and Hub Gene Identification

The STRING database (https://string-db.org/) was used to identify significant interactions among genes. DEGs were loaded, and interactions among them were extracted using a PPI interaction score threshold > 0.400 (22, 23). Subsequently, the numbers of nodes and edges were obtained using *Cytoscape software*, and the most important genes were identified using the cytoHubba tool in *Cytoscape*. Hub genes were identified based on degree centrality, representing key regulatory nodes in the morphine-induced molecular response.

## 4. Results

### 4.1. Gene Count Data Collection

RNA-Seq data were obtained from the study by Hofford et al. (14). As shown in Table 1, this analysis included 2 groups of mice (*Mus musculus*) from the NAc: a control group (n = 6) receiving saline injections and a treatment group (n = 5) receiving morphine (20 mg/kg for 7 days). Raw data, available under GEO Series accession GSE232836 and SRR accessions SRR24652590-SRR24652585 for saline and SRR24652578-SRR24652574 for morphine, were quality controlled and processed to generate gene count data for further analysis.

**Table 1.** RNA-Seq Sample Metadata for Mice Treated with Saline or Morphine

Series	SRR Accession	Treatment	Tissue	Organism	Platform
**GSE232836**			NAc	*Mus musculus*	Illumina HiSeq 2500
Control		Saline			
GSM7384342	SRR24652590				
GSM7384343	SRR24652589				
GSM7384344	SRR24652588				
GSM7384345	SRR24652587				
GSM7384346	SRR24652586				
GSM7384347	SRR24652585				
Morphine		Morphine 20 mg/kg for 7			
GSM7384354	SRR24652578				
GSM7384355	SRR24652577				
GSM7384356	SRR24652576				
GSM7384357	SRR24652575				
GSM7384358	SRR24652574				

### 4.2. Initial Results of Gene Count Data, PCA, Volcano Plot, DEG Identification, and Heatmap of Top DEGs

**Figure 1.**
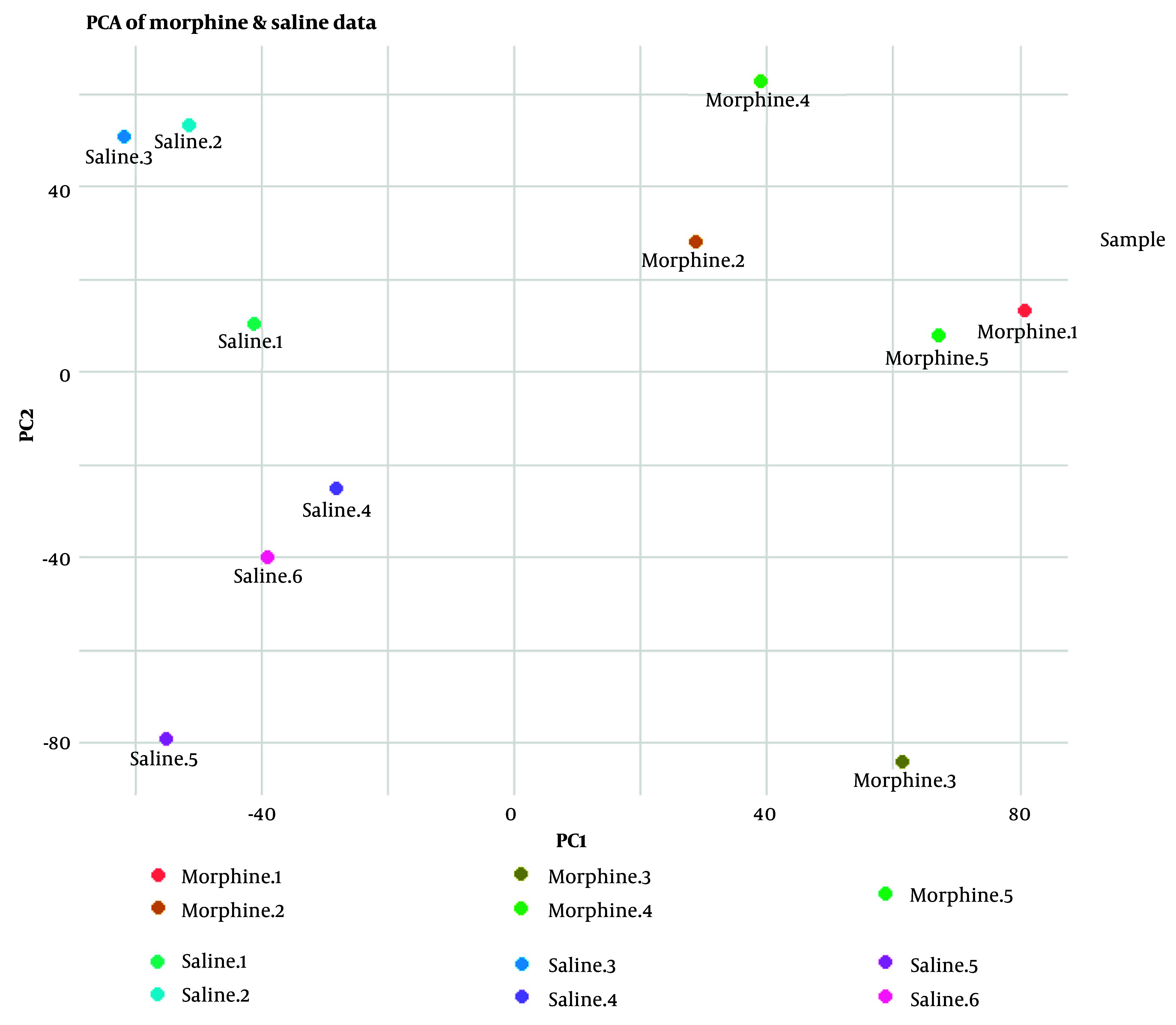
Principal component analysis of normalized gene expression, including log-transformed CPM, from NAc samples of morphine- versus saline-treated mice. PCA was performed in R using DESeq2, plyr, and ggplot2, and PC1 and PC2 are shown to visualize sample clustering based on global transcriptomic profiles. Morphine samples clustered separately from saline controls, indicating treatment-dependent transcriptomic changes.

The PCA plot showed clear separation between the morphine-treated and saline-treated groups along the principal components, indicating distinct transcriptomic profiles in the NAc following morphine exposure. Morphine samples clustered on the right and saline samples on the left, indicating distinct gene expression profiles between the 2 groups. Most saline samples clustered together, whereas morphine samples formed a separate cluster, suggesting that morphine administration induces substantial changes in gene expression. Some interindividual variability was observed within each group, as reflected by the spread of points within clusters. Separation along PC1 and PC2 indicates that the primary variance in the data is attributable to the treatment condition, morphine versus saline, rather than technical artifacts or batch effects (Figure 1).

**Figure 2.**
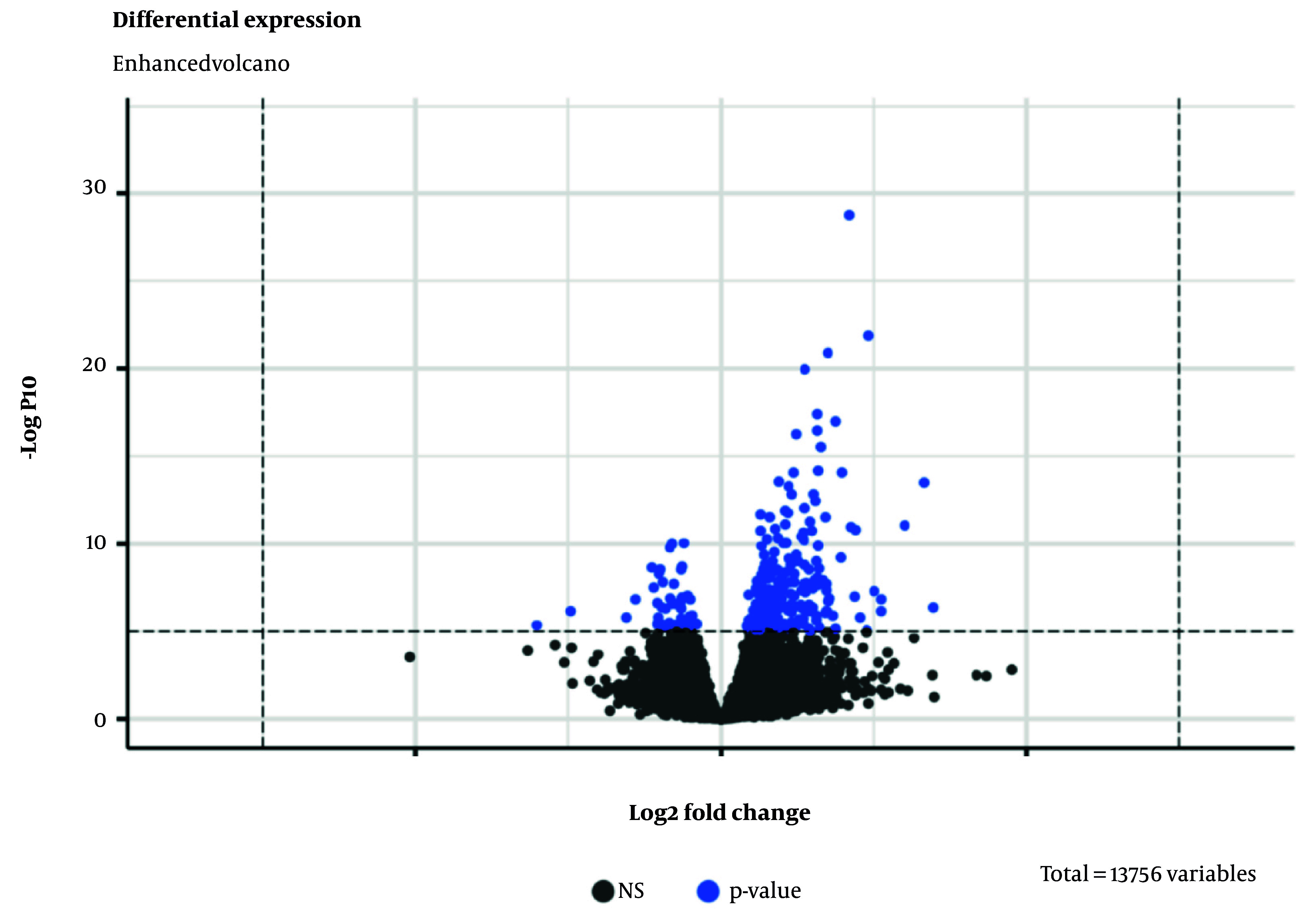
Volcano plot showing DESeq2-derived differential expression between morphine- and saline-treated NAc samples. Each point represents a gene plotted by log2 fold change on the x-axis and -log10 P value on the y-axis. Genes with P < 0.05 and log2 fold change > 0.5 were considered up-regulated, whereas genes with P < 0.05 and log2 fold change < -0.5 were considered down-regulated. Blue points indicate significantly up- or down-regulated genes, and gray points represent nonsignificant genes. The analysis identified 125 up-regulated and 18 down-regulated genes.

In the volcano plot (Figure 2), each point represents a gene plotted by log2 fold change on the x-axis and -log10 P value on the y-axis. Genes markedly up-regulated (log2 fold change > 0.5, P < 0.05) and down-regulated (log2 fold change < -0.5, P < 0.05) are shown in blue, whereas genes not meeting the significance thresholds are shown in gray. The volcano plot revealed a subset of genes with significant differential expression characterized by both high statistical significance and large fold changes. A total of 125 genes were up-regulated, and 18 genes were down-regulated.

**Figure 3.**
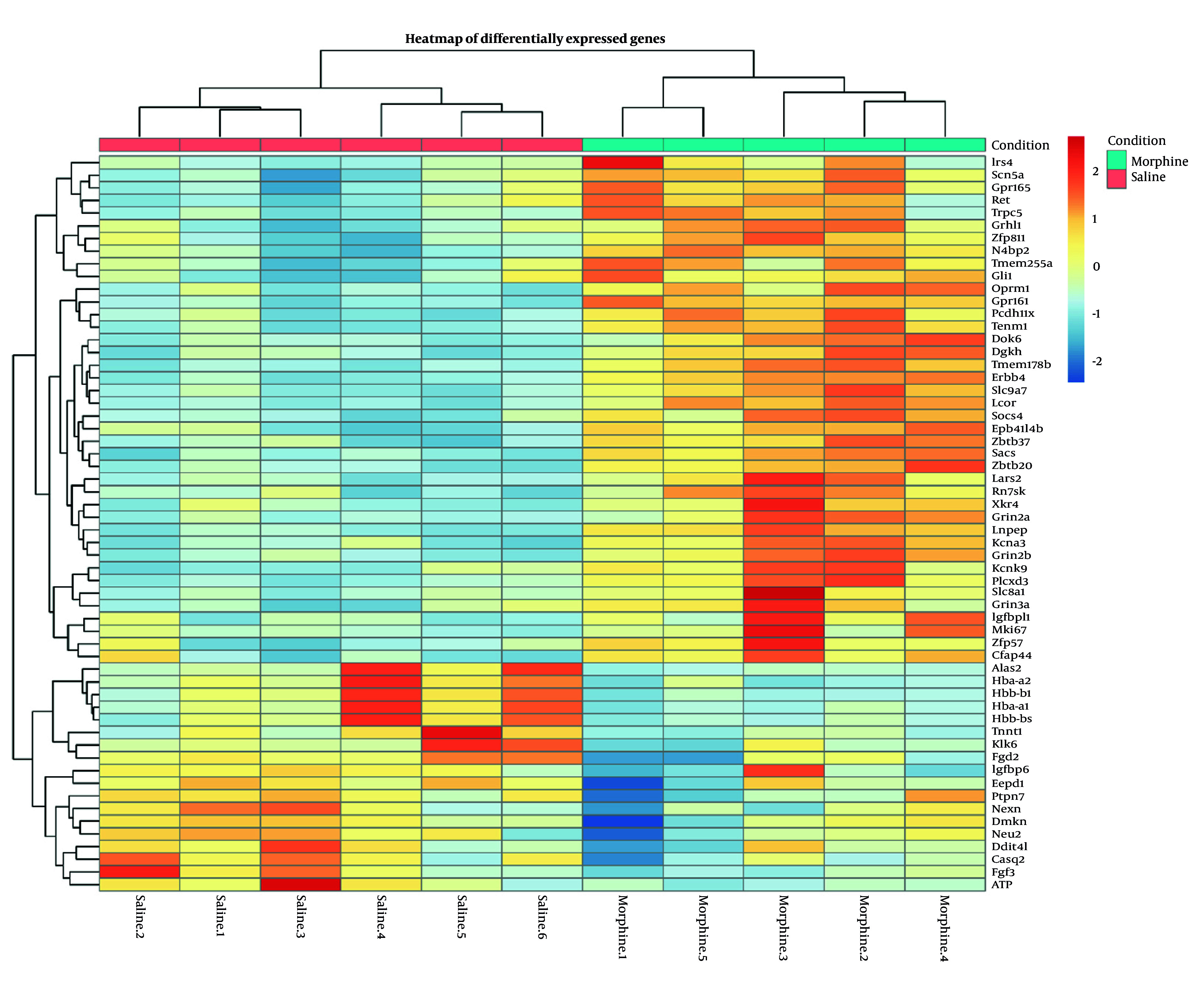
Heatmap of gene expression changes between morphine- and saline-treated NAc samples. Markedly up- and down-regulated genes identified from DESeq2 results were visualized using the pheatmap package in R. In the heatmap, rows represent genes and columns represent individual samples, and color intensity reflects relative expression levels. The heatmap clearly shows distinct expression patterns and robust segregation of morphine versus saline groups, with consistent up- or down-regulation of key genes, including fgf3, mki67, gli1, grin2a, ago2, ptch1, ret, erbb4, grin2b, kcna3, clcn5, and alas2.

In the heatmap of expression levels for markedly up- and down-regulated genes (Figure 3), rows represent genes and columns represent individual samples from the morphine and saline groups. Color intensity reflects relative expression levels, illustrating distinct expression patterns between groups. In this figure, differences in the expression of up- and down-regulated genes are clearly visible. Differences in color indicate changes in gene expression. Heatmap visualization of the most significantly altered genes showed robust segregation between the morphine and saline groups. Key genes, such as fgf3 (fibroblast growth factor 3), mki67 (marker of proliferation Ki-67), gli1 (GLI family zinc finger 1), grin2a (glutamate receptor NMDA subunit 2A), ago2 (argonaute RISC catalytic component 2), ptch1 (patched 1), ret (ret proto-oncogene), erbb4 (erb-b2 receptor tyrosine kinase 4), grin2b (glutamate receptor NMDA subunit 2B), kcna3 (potassium voltage-gated channel subfamily A regulatory beta subunit 3), clcn5 (chloride voltage-gated channel 5), and alas2 (aminolevulinate synthase 2), exhibited consistent up- or down-regulation patterns, highlighting their potential roles in the morphine response.

### 4.3. GO and KEGG Enrichment

To enhance our understanding, we analyzed the up- and down-regulated genes identified using the DESeq2 package in R and used GO to categorize these genes by biological processes, cellular components, and molecular functions, as illustrated in 3 figures.

**Figure 4.**
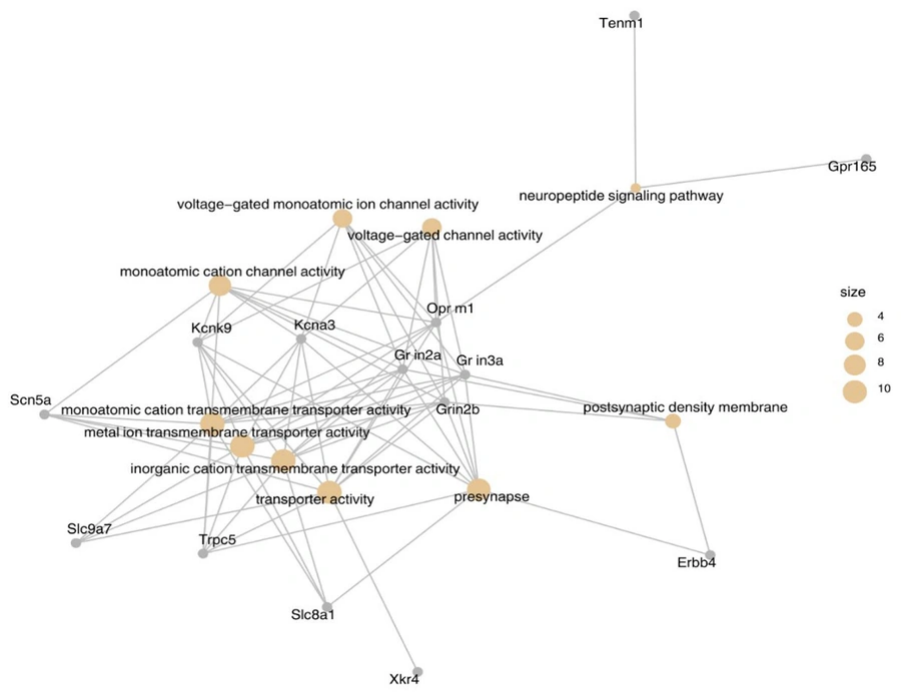
Gene Ontology category network plot (cnetplot) illustrating associations between differentially expressed genes and enriched GO terms. GO enrichment was performed in R using clusterProfiler with org.Mm.eg.db from Bioconductor. In the network, nodes represent genes and GO terms, and edges indicate gene-term relationships. Key hub genes, including grin2a, grin2b, oprm1, and kcna3, participate in multiple functional categories, highlighting their central involvement in neuronal signaling and synapse-related processes.

The cnetplot (Figure 4) visualizes the connections between key genes and enriched GO terms. Nodes represent genes and GO terms, whereas edges indicate gene-term associations. This network highlights categories such as voltage-gated monoatomic ion channel activity, transporter activity, and postsynaptic density membrane. Hub genes, including grin2a, grin2b, oprm1 (opioid receptor mu 1), and kcna3, participated in multiple functional categories, underscoring their central roles in neuronal signaling.

**Figure 5.**
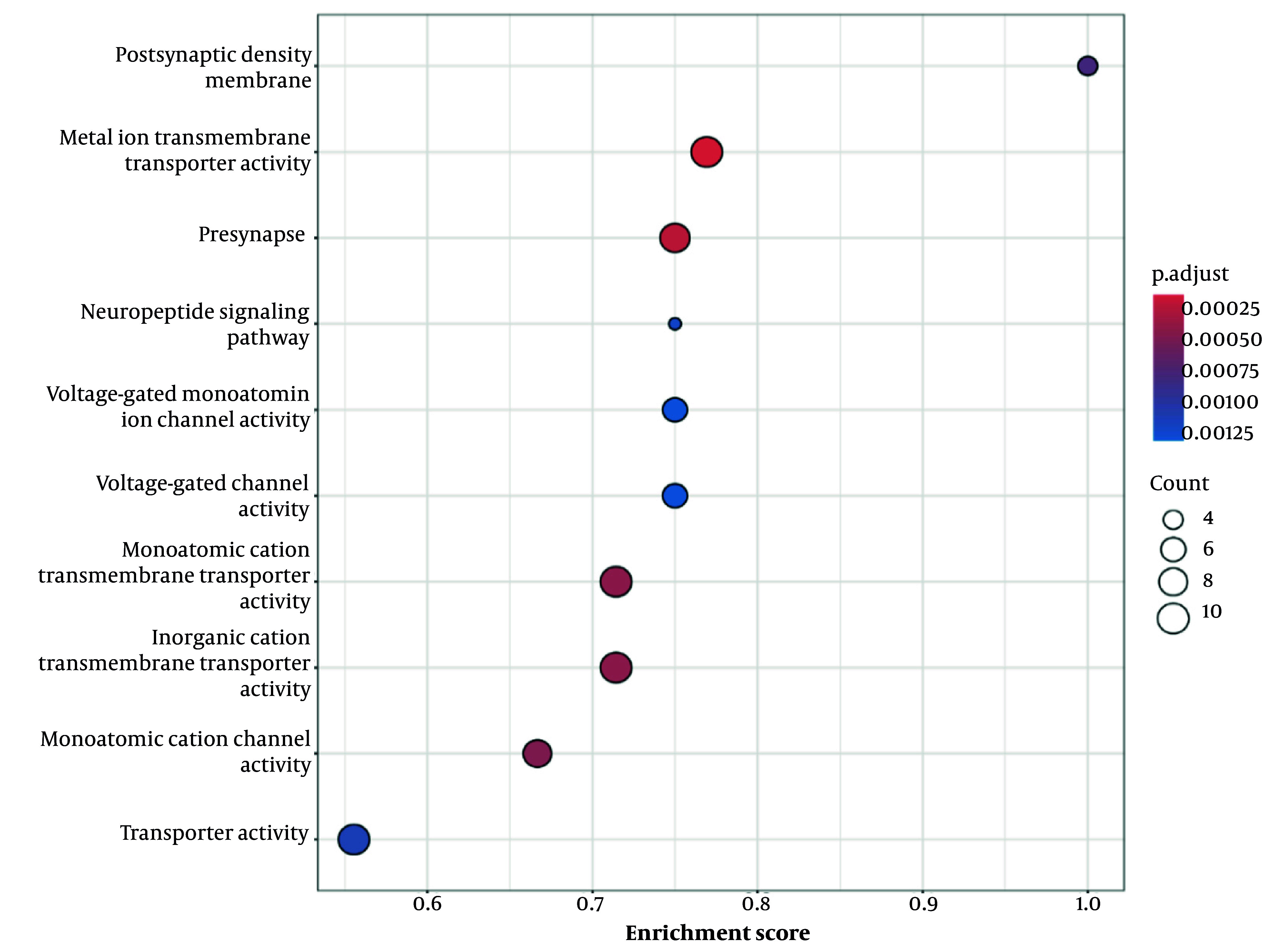
Dotplot of GO enrichment results for up- and down-regulated DEGs identified by DESeq2. The y-axis lists enriched GO terms, and the x-axis shows the enrichment score. Each dot represents a GO term, with dot size proportional to the number of associated genes and color indicating the adjusted P value (P.adjust), where more significant terms are highlighted. Enriched categories mainly relate to synaptic membrane components, transporter activity, and presynaptic structures.

Figure 5 illustrates the results of GO enrichment analysis and shows the top-ranked GO terms associated with the dataset under study. The y-axis lists GO terms, such as postsynaptic density membrane, metal ion transmembrane transporter activity, and presynapse. The x-axis displays the enrichment score, which quantitatively reflects the degree of overrepresentation of each GO term within the analyzed gene list. Each dot denotes a specific GO term, with dot size corresponding to the gene count annotated to that term. The color gradient indicates the adjusted P value (P.adjust), with redder hues indicating lower and more significant P values after multiple-testing correction, reflecting stronger statistical significance. This analysis highlights several significantly enriched terms related to synaptic membrane components and transporter activities. Notably, terms such as postsynaptic density membrane, metal ion transmembrane transporter activity, and presynapse had high enrichment scores and strong statistical significance. These findings suggest pronounced involvement of these functional categories in the biological mechanisms characterized by the studied gene set.

**Figure 6.**
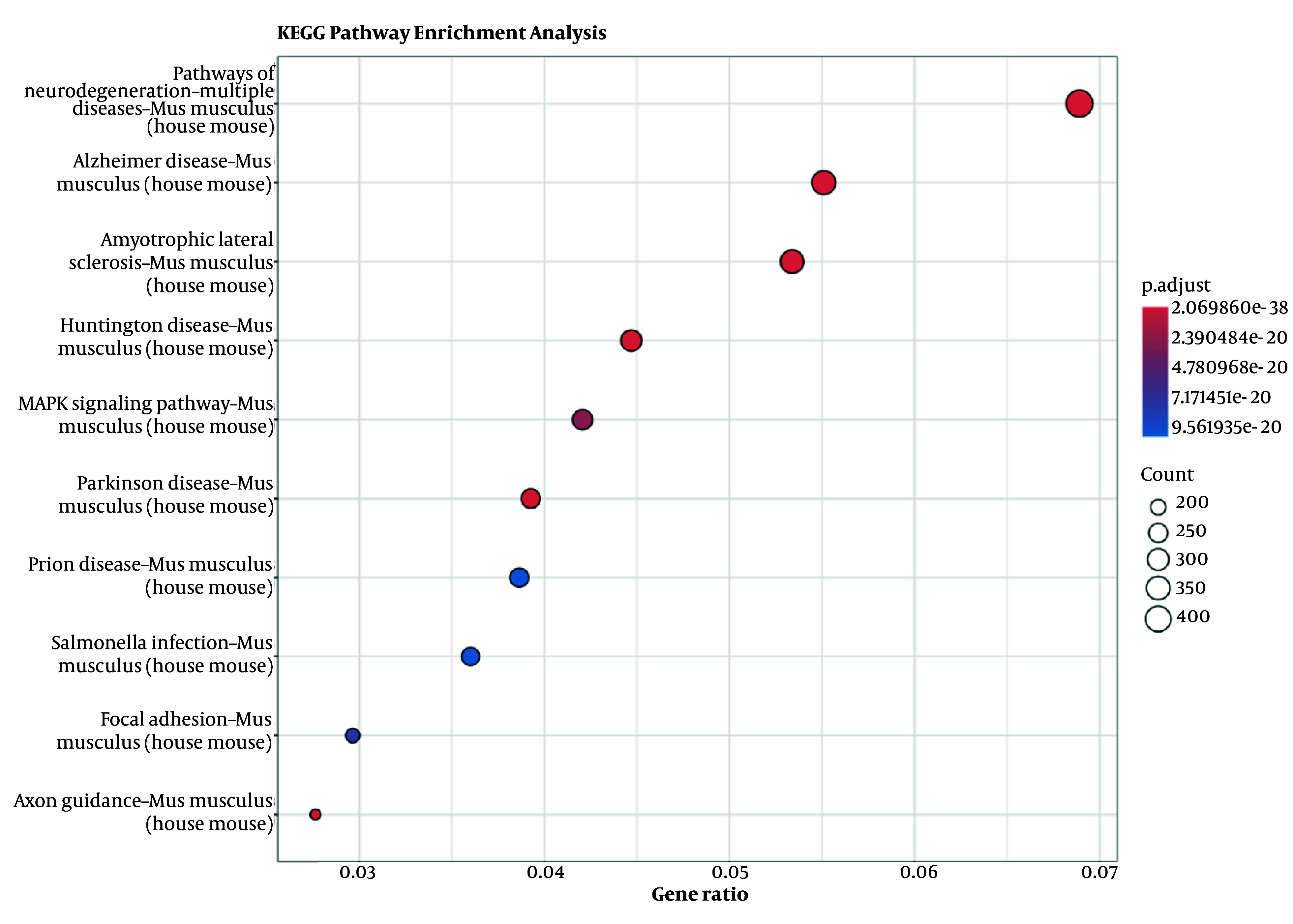
Kyoto Encyclopedia of Genes and Genomes pathway enrichment dotplot comparing morphine-treated and control groups. The y-axis shows enriched pathways, and the x-axis displays the gene ratio, defined as the DEG proportion per pathway. Dot size corresponds to the number of mapped genes, and dot color reflects adjusted P value significance. Morphine exposure significantly enriched pathways involved in neurodegeneration and synaptic signaling, including neurodegenerative disease-associated pathways such as Alzheimer disease, amyotrophic lateral sclerosis, and Huntington disease.

Figure 6 shows significantly enriched KEGG pathways in the morphine-treated group compared with controls. The y-axis lists enriched pathways, and the x-axis shows the gene ratio, defined as the proportion of DEGs involved in each pathway relative to the total genes annotated to that pathway. Dot size indicates the number of genes mapped to each pathway, and dot color intensity corresponds to statistical significance based on the adjusted P value, with darker colors indicating higher significance. Figure 6 revealed that the DEGs were significantly enriched in pathways related to neurodegeneration and synaptic signaling, including pathways of neurodegeneration and neurodegenerative diseases such as Alzheimer disease, amyotrophic lateral sclerosis, and Huntington disease. These findings suggest that morphine exposure induces broad transcriptional changes affecting synaptic organization and neurodegenerative processes.

### 4.4. PPI Network and Hub Gene Analysis

**Figure 7.**
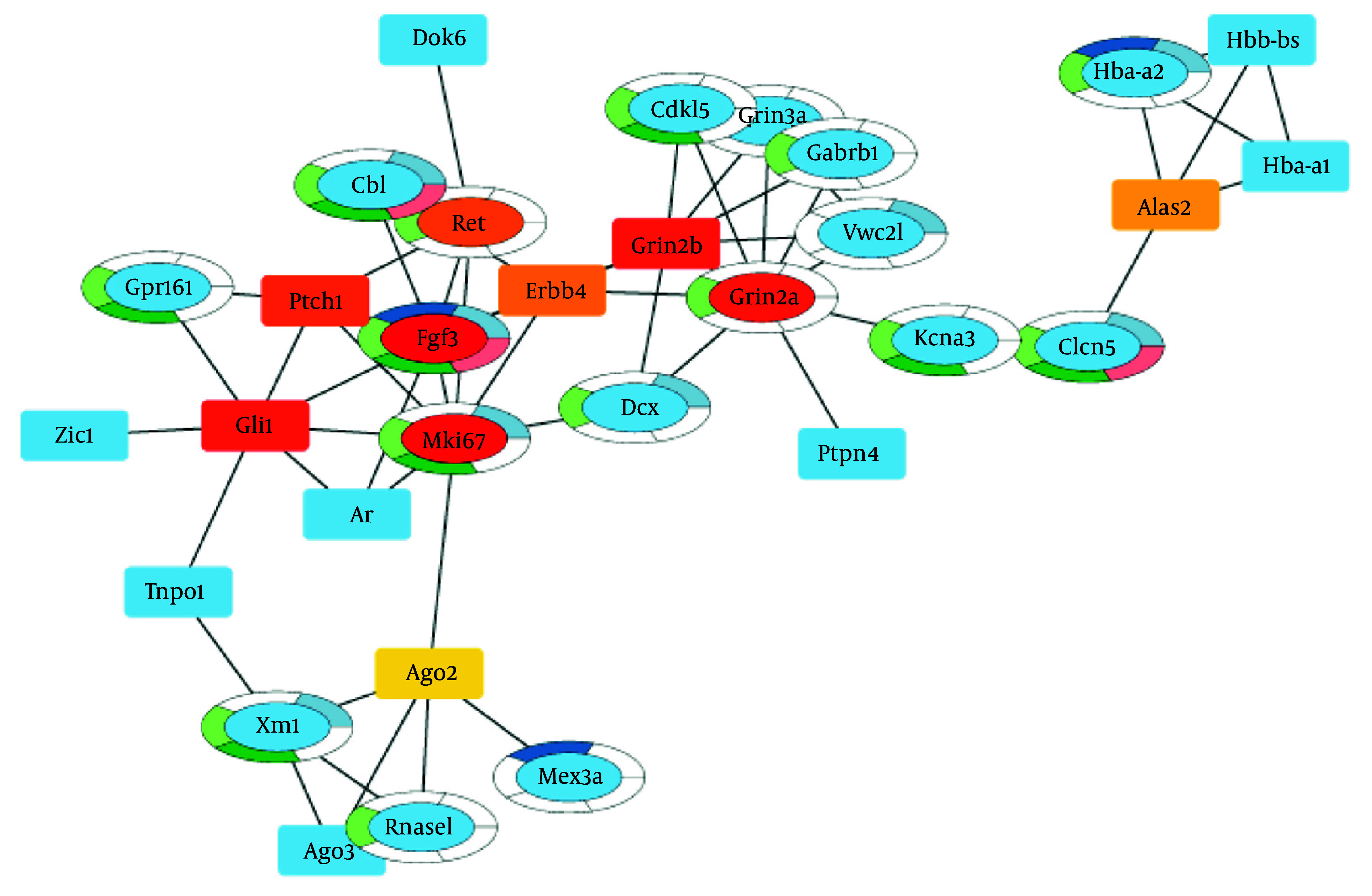
Protein-protein interaction network of DEGs associated with morphine treatment. The network was constructed using the STRING database and visualized in *Cytoscape*. Interactions with a score > 0.400 were included, resulting in 77 nodes and 95 edges. Hub genes, identified by the cytoHubba tool based on degree centrality, are highlighted and include fgf3, mki67, grin2b, grin2a, gli1, ptch1, ret, erbb4, alas2, and ago2. These hub genes are proposed to be critical regulators mediating neuroplasticity, synaptic transmission, and epigenetic regulation in response to morphine.

Protein-protein interactions were analyzed using the STRING database and Cytoscape software. The Cytoscape output revealed 77 nodes and 95 edges. Using the cytoHubba tool in *Cytoscape*, we identified the hub genes shown in Figure 7. These genes included fgf3, mki67, grin2b, grin2a, gli1, ptch1, ret, erbb4, alas2, and ago2. These hub genes likely serve as critical regulators of molecular adaptations to morphine, mediating neuroplasticity, synaptic transmission, and epigenetic regulation.

## 5. Discussion

The prevalence of OUD is increasing worldwide and is accompanied by escalating fatal outcomes, highlighting the urgent need to improve treatment access, prevention, and harm-reduction strategies globally (2, 24). The chronic, relapsing nature of OUD, together with reduced quality of life, underscores the need for enhanced intervention strategies, broader access to effective therapies, and comprehensive public health approaches to reduce both individual and societal harms related to OUD (25-27). Morphine, although widely used for its potent analgesic properties, poses a significant risk of dependence, leading to persistent neurobiological changes in key brain regions such as the NAc, an important area of the reward system (28, 29). Chronic morphine exposure induces neuroplasticity that underlies tolerance, dependence, and withdrawal (30-33). Unraveling the molecular basis of these changes is challenging because of the complexity of the underlying pathways. In recent years, bioinformatics has emerged as a powerful tool to address these challenges, enabling the integration, analysis, and interpretation of high-throughput datasets to provide mechanistic insights into substance use disorders (10, 34). In the present study, we applied RNA-Seq and comprehensive bioinformatics analyses to characterize morphine-induced gene expression changes in the NAc of mice, aiming to identify key molecular players and pathways involved in morphine-induced alterations.

Studies have shown that addictive drugs, such as opioids including morphine, induce changes in the reward system and the NAc (35, 36). Investigating cellular and molecular changes in the NAc can improve understanding of the mechanisms of morphine addiction. The objective of this study was to identify novel associations between gene expression changes in the morphine-treated group and those in the control group using various bioinformatics and data-analysis methods, including the DESeq2 package in R, GO, and pathway enrichment analysis.

The clear segregation of the morphine and saline groups in PCA underscores the robustness of morphine-induced transcriptomic alterations in the NAc. Principal component analysis is widely used to assess sample quality and identify major drivers of variance in RNA-Seq datasets (37), confirming that morphine treatment is the primary source of variation in this study.

The volcano plot effectively visualized the landscape of gene expression changes, identifying genes with both statistical significance and biologically meaningful fold changes (38). These genes represent candidates for further mechanistic studies. Hierarchical clustering in the heatmap demonstrated distinct expression signatures between the morphine and control groups. The PPI network also highlighted hub genes that likely orchestrate complex molecular responses to morphine. These integrated transcriptomic and network analyses reveal a coherent molecular framework underlying morphine-induced adaptations in the NAc. These genes include fgf3, a member of the fibroblast growth factor family that mediates growth, development, and differentiation (39); mki67, a proliferation marker (40-43); and NMDA receptor subunits grin2a and grin2b, which are known mediators of synaptic plasticity and opioid-induced neuroadaptations (44-47). Moreover, the involvement of hedgehog signaling components, such as gli1 and ptch1, aligns with reports linking this pathway to opioid tolerance (48, 49). In addition to the proteins mentioned above, these hubs also include erbb4, ret, alas2, and ago2 (50-52).

Central to these findings is the convergence of multiple key pathways and gene networks that collectively shape the neurobiological response to morphine. Notably, genes encoding NMDA receptor subunits, including grin2a and grin2b, emerge as critical mediators of synaptic plasticity, a fundamental process in addiction-related learning and memory (53-58). Their prominent role aligns with enriched glutamatergic synapse pathways, indicating that morphine profoundly modulates excitatory neurotransmission within the NAc (59).

Furthermore, factors such as fgf3 (60), erbb4 (61-63), and ret (64) regulate neuronal survival and synaptic remodeling, highlighting the brain’s adaptive capacity in response to morphine-induced stress. The up-regulation of mki67 (42, 43, 65) also suggests that morphine may influence cellular proliferation or neurogenesis, extending its impact beyond synaptic modulation to alterations in cellular composition and plasticity within the reward circuitry.

Activation of the sonic hedgehog signaling pathway, indicated by altered expression of gli1 and ptch1 (66, 67), further underscores the engagement of developmental and regenerative mechanisms by morphine. The involvement of this pathway suggests that morphine triggers complex molecular programs that contribute to both adaptive and maladaptive neural remodeling.

Crucially, the identification of ago2, a key regulator of gene silencing, highlights the role of epigenetic and post-transcriptional regulation in sustaining the effects of morphine. This finding suggests stable, heritable changes in gene expression that underpin the long-lasting behavioral adaptations characteristic of addiction (52, 68). Additional hub genes related to ion-channel function and cellular metabolism, such as kcna3 (69) and alas2, respectively, emphasize the importance of maintaining neuronal excitability and metabolic homeostasis during morphine exposure.

Together, these interconnected molecular themes indicate that morphine orchestrates a multifaceted neurobiological response in the NAc, integrating synaptic plasticity, growth-factor signaling, neurogenesis, developmental pathways, and epigenetic regulation. This integrated network not only mediates the acute pharmacological effects of morphine but also drives the enduring neuroadaptations that underlie addiction vulnerability, offering valuable targets for therapeutic intervention. In addition, the hub genes identified in this study, such as grin2a and grin2b, not only reflect mechanistic signatures of morphine-induced neuroadaptations but also represent promising candidates for biomarker development in OUD. Such biomarkers may be helpful for the early detection of vulnerability, monitoring treatment efficacy, and predicting relapse risk. However, further investigations are needed to validate these candidates at the clinical stage.

This study provides comprehensive transcriptomic insights into morphine-induced molecular adaptations in the mouse NAc. The identification of key DEGs, enriched biological pathways, and hub genes underscores the complexity of morphine-induced neuroplasticity. These findings pave the way for targeted investigations into the roles of fgf3, gli1, NMDA receptor subunits, and epigenetic regulators in morphine addiction mechanisms.

Future research should focus on functional validation of these hub genes and pathways using in vivo and in vitro models to determine their causal roles in morphine-induced behavioral and cellular changes. Moreover, exploring therapeutic interventions that modulate these molecular targets may yield novel strategies for treating OUD. However, it is important to recognize that transcriptomic studies of opioid addiction often face limitations, such as sample size, phenotypic heterogeneity, and a lack of longitudinal data, which may affect the generalizability and robustness of the findings. Moreover, because addiction is a complex brain disorder often accompanied by behavioral disturbances, conducting translational studies directly in humans remains challenging.

### 5.1. Conclusions

This study employed RNA-Seq and comprehensive bioinformatics analyses to elucidate molecular adaptations in the mouse NAc following chronic morphine exposure. Our findings reveal significant transcriptomic alterations characterized by the identification of DEGs, enriched biological pathways, and crucial hub genes. Notably, we observed pronounced modulation of pathways related to synaptic plasticity, neuronal signaling, and neurodevelopmental processes, including the significant involvement of NMDA receptor subunits, such as grin2a and grin2b, fibroblast growth factor family member 3 (fgf3), and hedgehog signaling components, such as gli1 and ptch1. The identified hub genes, including fgf3, mki67, grin2a, grin2b, gli1, ptch1, ret, erbb4, alas2, and ago2, likely orchestrate the complex neurobiological response to morphine, mediating adaptations that underlie tolerance, dependence, and potentially long-lasting vulnerability to OUD.

The importance of this study lies in its contribution to a deeper understanding of the intricate molecular mechanisms driving OUD. By identifying key genes and pathways, our research offers potential novel targets for therapeutic interventions aimed at treating opioid addiction. The identified biomarkers, particularly genes such as grin2a and grin2b, may also hold promise for future development in early detection, treatment monitoring, and relapse prediction.

Future research should prioritize functional validation of these identified hub genes and pathways using in vivo and in vitro experimental models to establish their causal roles in morphine-induced behavioral and cellular changes. Investigating therapeutic strategies that target these molecular players could lead to the development of more effective treatments for OUD. Furthermore, translational studies are needed to validate these findings in human cohorts and explore the potential of these molecular signatures as clinical biomarkers. Addressing the inherent challenges in addiction research, such as sample heterogeneity and the complexity of clinical translation, will be crucial for advancing the field.

## Data Availability

The dataset presented in the study is available on request from the corresponding author during submission or after publication. The data are not publicly available due to privacy concerns and consent restrictions.
